# The storage time of cryopreserved human spermatozoa does not affect pathways involved in fertility

**DOI:** 10.1186/s12610-024-00231-4

**Published:** 2024-09-17

**Authors:** Sara Stigliani, Adriana Amaro, Francesco Reggiani, Elena Maccarini, Claudia Massarotti, Matteo Lambertini, Paola Anserini, Paola Scaruffi

**Affiliations:** 1 SS Physiopathology of Human Reproduction, IRCCS Ospedale Policlinico San Martino, Genova, Italy; 2 SSD Regolazione dell’Espressione Genica, IRCCS Ospedale Policlinico San Martino, Genova, Italy; 3 Department of Neuroscience, Rehabilitation, Ophthalmology, Genetics and Maternal-Child Health (DiNOGMI), University of Genova, Genova, Italy; 4 Department of Internal Medicine and Medical Sciences (DiMI), University of Genova, Genova, Italy; 5 UOC Clinica di Oncologia Medica, IRCCS Ospedale Policlinico San Martino, Genova, Italy; 6 SS Physiopathology of Human Reproduction, IRCCS Ospedale Policlinico San Martino, Largo R. Benzi, 10, Genova, 16132 Italy

**Keywords:** Human sperm, Cryopreservation, Storage time, Microarray, Transcriptome, Fertility preservation, Spermatozoïdes humains, Cryoconservation, Temps de stockage, Puces à ADN, Transcriptome, Préservation de la fertilité

## Abstract

**Background:**

Cryopreservation of human spermatozoa is a widely used technique in the assisted reproduction technology laboratory for the storage of gametes for later use, for the fertility preservation and for sperm donation programs. Cryopreservation can cause damage to membrane, cytoskeletal, acrosome and increased oxidative stress, sperm DNA damage and transcriptome changes. To assess the impact of storage time on the transcriptome of frozen human spermatozoa, semen samples were collected from 24 normospermic donors of whom 13 had cryostored semen for a short-time (1 week) and 11 had cryostored semen for a long-time (median 9 years).

**Results:**

RNA was extracted from each frozen-thawed sperm sample, randomized in pools, and analyzed by microarrays. Five transcripts were in higher abundance in the long-time respect to the short-time storage group. Functional annotation enrichment disclosed that that the length of cryostorage has no effect on critical pathways involved in sperm physiology and function.

**Conclusions:**

The storage time of cryopreserved human spermatozoa does not affect pathways involved in fertility.

**Supplementary Information:**

The online version contains supplementary material available at 10.1186/s12610-024-00231-4.

## Résumé

**Contexte:**

La cryoconservation des spermatozoïdes humains est une technique largement utilisée, dans les laboratoires de procréation médicalement assistée, pour le stockage des gamètes en vue d’une utilisation ultérieure, dans le cadre d’une préservation de la fertilité et dans les programmes de don de sperme. La cryoconservation peut altérer la membrane, le cytosquelette, l’acrosome, et augmenter le stress oxydatif des spermatozoïdes, endommager l’ADN et modifier le transcriptome. Pour évaluer l’impact du temps de stockage sur le transcriptome de spermatozoïdes humains congelés, des échantillons de sperme ont été prélevés auprès de 24 donneurs normozoospermiques, dont 13 avaient cryoconservé du sperme pendant une courte période (1 semaine) et 11 avaient cryoconservé du sperme pendant une longue période (médiane de 9 ans).

**Résultats:**

L’ARN a été extrait de chaque échantillon de sperme congelé-décongelé, randomisé dans des pools et analysé par microarrays. Cinq transcrits étaient en plus grande abondance dans le groupe de stockage à long terme que dans le groupe de stockage de courte durée. L’enrichissement en annotation fonctionnelle a révélé que la durée de la cryoconservation n’a aucun effet sur les voies critiques impliquées dans la physiologie et la fonction des spermatozoïdes.

**Conclusions:**

Le temps de stockage des spermatozoïdes humains cryoconservés n’affecte pas les voies impliquées dans la fertilité.

## Background

Cryopreservation of human spermatozoa in liquid nitrogen is a widely used technique in the assisted reproduction technology (ART) laboratory for the storage of gametes for later use, for the fertility preservation (i.e. men undergoing gonadotoxic therapies or surgery), and for sperm donation programs. Although most spermatozoa retain their motility, viability and fertilization potential following the freeze-thaw process, the cryopreservation technique can cause damage to membrane, cytoskeletal, acrosome [1] and increased oxidative stress [2]. Cryopreservation is also associated with sperm DNA damage and transcriptome changes [3–5].

The long-term viability of frozen semen is especially important for young men, who had performed sperm cryopreservation for gonadotoxic treatments, as there may be many years between the time when semen is frozen and when it is used in ART cycles.

Clinical successes with human sperm cryopreserved prior to cancer treatment and long-term banked have been reported: case reports describe live births from ICSI (intracytoplasmatic sperm injection) with semen stored for 21 [6] and 40 [7] years, and from intrauterine insemination (IUI) with semen stored for 21 and 28 years [8]. A recent systematic review and meta-analysis evaluated the influence of long-term cryostorage on human sperm quite reassuring that sperm cryopreservation does not adversely affect post-thaw clinical and obstetric outcomes [9].

From a biological point of view, the sperm could theoretically be cryostored indefinitely or at least for a very long period. Functional tests revealed that long-term cryostored sperm retains a good recovery of post-thaw motility concentration [10], including progressive motility concentration [11], normal levels of binding to the human zona pellucida and zona-induced acrosome reaction [10]. It was also reported that long-term cryostorage of donor sperm did not affect DNA integrity any more than short-term cryostorage did [12]. Conversely, some cryoinjuries may occur later in the freezing process: cryopreservation of human semen not only causes damage to different cellular levels, but also has a time-dependent effect at the level of cytoskeletal [13].

Increased reactive oxygen species (ROS) production during freezing–thawing process can cause DNA damage, alterations in DNA methylation, epigenetic instability, including increased alternative splicing events and changes in crucial mitochondrial functional activities [14, 15]. There is little information about the effects of cryopreservation on epigenetic modulation in sperm and the health of children born with frozen spermatozoa [16].

Overall, the effect of storage time on the biology of frozen human sperm is still an inconclusive topic and no data on molecular profiles are available so far. The aim of this study was to investigate whether the storage time modified the gene expression profile of cryopreserved human sperm.

## Methods

### Study design and setting

This study included 24 normozoospermic men, of whom 13 had cryostored semen for a short-time (1 week) and 11 had cryostored semen for a long-time (median 9 years, range 7–10). The short-time group included semen samples that were donated for this study and were cryopreserved for this purpose. The long-time group included semen samples that were banked at our center and donated for research. Supplementary Table 1 shows the semen parameters and clinical data of donors enrolled in the study.

### Participants

**Fig. 1:**
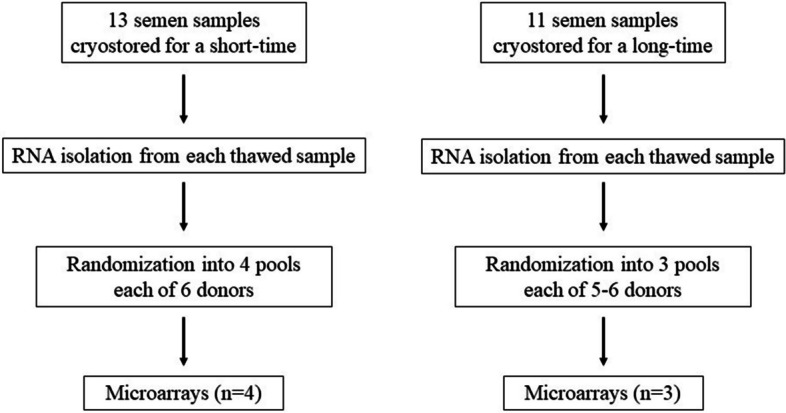
Flowchart of the study. Legend. The Figure shows the different task of the study protocol

The men enrolled in this study had a median age of 34.5 years (range: 22.0–46.0). The age of men was similar in the two groups (short-time group: median age = 35 years, interquartile range: 33.7–42.5; long-time group: 31 years, interquartile range: 26.0–38.0; *p* = 0.1473). RNA was extracted from each frozen-thawed sperm sample and randomized into pools (4 pools for short-time samples and 3 pools for long-time samples), each of 5–6 donors, in order to minimize any donor-specific variability in gene expression. Each donor was randomized in 2 pools. Each pool was not tested in replicate because the amount of RNA available for samples was a technical limitation. Wide-transcriptome analysis of short- and long-time pools was performed. Figure 1 shows the flowchart of the study.

### Sperm cryopreservation and thawing

After collection by masturbation, sperm samples were liquefied at room temperature for 30–60 min. The SpermFreeze™ medium (FertiPro NV, Beernem, Belgium) was added to the sperm in drops while gently swirling (0.7 ml of medium per ml of sperm). During the 10 min incubation for equilibration at room temperature, the mixture was sucked into the CBS High Security sperm straws (Cryo Bio System, L'Aigle, France) that were placed in liquid nitrogen vapor phase for 15 min. Then the straws were quickly transferred to liquid nitrogen and stored at -196 °C.

To thaw, the straws were removed from the liquid nitrogen and warmed at room temperature until the sample thawed. Then the end of the straw was cut off and the semen-medium mixture was put into a tube containing 1 ml of Gamete buffer (Cook Medical) prewarmed at 37 °C. After centrifugation at 1400 rpm for 10 min the pellet was transferred into the QIAzol® Lysis Reagent (Qiagen) and processed as described below.

### RNA isolation and quantification

Total RNA was extracted using the miRNeasy Micro kit (Qiagen), according to the manufacturer’s procedure. High Sensitivity RNA kit on 2200 Tape Station system (Agilent Technologies, Santa Clara, CA, USA) was used to quantify and control the quality of RNAs. The RNA samples were randomized in pools.

### Gene expression profiling

Five ng of total RNA from each pool were amplified using Ovation Pico WTA System V2 (NuGEN Technologies, San Carlos, CA, USA) and labeled by Enzymatic Labeling Kit (Agilent Technologies). Three µg of purified-Cye3-labeled cDNA were hybridized to Human GE 4 × 44 K v2 microarrays (Agilent Technologies) at 65 °C, 10 rpm for 17 h. Slides were washed and scanned by Agilent G2505C scanner. Data were extracted using Feature Extraction (FE) software v10.7, GE1_1100_Jul11 protocol (Agilent Technologies). Microarray raw data have been deposited in the National Center for Biotechnology Information Gene Expression Omnibus (GEO, http://www.ncbi.nlm.nih.gov/geo/) and are accessible through GEO access number GSE225320.

### Statistical analysis of microarray data

Tab-delimited text files containing FE results were acquired. The unpaired Significance Analysis of Microarrays (SAM) was performed in R/BioConductor using the limma R package to normalize data, performing background correction and quantile normalization between arrays. Array probe annotation was performed with hgug4112a.db and samr R packages Differentially expressed genes were identified by applying variance and intensity filters. Significant genes (probes with False Discovery Rate (FDR) = 0) were clustered by hierarchical clustering with average linkage and Euclidean distance measure. Probes without annotation, low counts or without annotations have been removed.

### Functional annotation and Gene Ontology analysis

Functional enrichment annotation analysis of the Gene Ontology (GO) categories was performed using the Database for Annotation, Visualization, and Integrated Discovery (DAVID) Bioinformatics Resources (https://david.ncifcrf.gov/) [17, 18] and the Kyoto Encyclopedia of Genes and Genomes (KEGG) [19] to identify pathways significantly (*P* < 0.05) over-represented in our datasets.

## Results

**Fig. 2:**
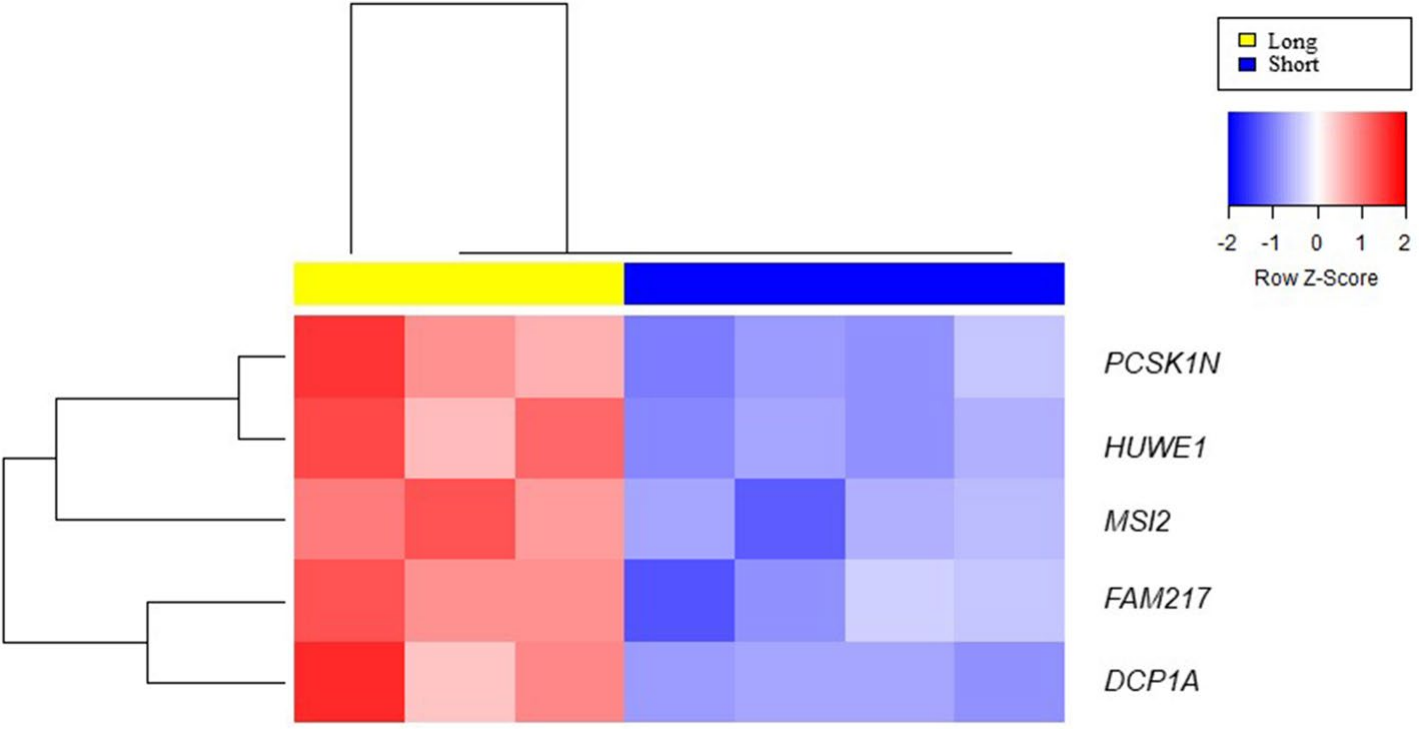
Agglomerative hierarchical clustered heat map of differentially expressed genes in short- *versus* long-time storage sperm. Legend: Short -time storage sperm samples are indicated as “short” in blue; long-time storage sperm samples are indicated as “long” in yellow. Each color patch represents the amount of transcripts (row) in that sample (column), with a continuum of levels from bright blue (lowest) to bright red (highest). Significant genes (probes with False Discovery Rate = 0) were clustered with average linkage and Euclidean distance measure

Comparison of gene expression profiles between thawed sperm after a short- or long-time storage in liquid nitrogen identified 5 unique transcripts more abundant in the latter group (*DCP1A*, *FAM217A*, *HUWE1*, *MSI2*, *PCSK1N*) (Fig. 2, Table 1).

**Table 1:** Fold changes of the 5 transcripts identified in the comparison between short-time and long-time groups

Gene	Fold change
*DCP1A*	5.019092
*FAM217A*	4.778052
*HUWE1*	4.893018
*MSI2*	5.071137
*PCSK1N*	5.656292

The Table shows the fold changes of the transcripts more abundant in the long-time respect to the short-time storage group

To get an insight into their potential functional role, the five genes differentially expressed by the two groups were annotated for GO terms including molecular function, biological process, and cellular components. Functional annotation enrichment has shown that the 5 transcripts more abundant in the long-time stored sperm are involved in methylation and mRNA binding (Table 2), ubiquitin mediated proteolysis, RNA degradation and surveillance pathways (Table 3). They were located in the cytoplasm at the level of nucleus (*DCP1A*, *FAM217A, HUWE1, MSI2*), mitochondria (*HUWE1*), Golgi apparatus and endoplasmic reticulum (*PCSK1N*).

**Table 2:** The most significantly enriched categories in the transcripts more abundant in semen cryostored for long-time

Category	Term	*P* value	Genes
UP_KW_PTM	KW-0488 ~ Methylation	1.6 E-02	*DCP1A*, *HUWE1*, *MSI2*
GOTERM_CC_DIRECT	GO:0005829 ~ cytosol	1.8 E-01	*DCP1A*, *HUWE1*, *MSI2*
GOTERM_CC_DIRECT	GO:0005737 ~ cytoplasm	1.9 E-01	*DCP1A*, *HUWE1*, *MSI2*
GOTERM_MF_DIRECT	GO:0003729 ~ mRNA binding	5.3 E-02	*DCP1A*, *MSI2*

The table shows the most significantly (*P* < 0.05) enriched categories for cellular components (*CC*) and molecular function (*MF*) terms in the 5 transcripts more abundant in semen cryostored for a long-time respect to semen cryostored for a short-time. Fisher’s Exact test was used to measure the gene-enrichment in annotation terms

**Table 3:** Functional annotation of the transcripts more abundant in semen cryostored for a long-time respect to semen cryostored for a short-time according to KEGG pathway database

**Entry number**^a^	**Name**^b^	**Genes**
hsa04120	Ubiquitin mediated proteolysis	*HUWE1*
hsa03018	RNA degradation	*DCP1A*
hsa03015	mRNA surveillance pathway	*MSI2*

The Table shows the pathways associated to three of the five transcripts more abundant in semen cryostored for a long-time respect to semen cryostored for a short-time

^a^Entry of each pathway identified by a five-digit number preceded by three- letter organism code (has = *homo sapiens*)

^b^The name of the pathway

## Discussion

Nowadays, cryopreservation of sperm is widespread in human ART for several clinical, logistic and social issues, including fertility preservation before gonadotoxic treatments or surgery or in the case of medical conditions that could compromise fertility, storage of gametes for later use, cryobanking for sperm donation programs. The efficiency of the procedure has been dramatically increased in the past decade, and it often implies a long-term cryostorage of frozen sperm sample before its use.

It is widely accepted that mature spermatozoa are both transcriptionally and translationally silent and that cryopreservation can lead to changes in transcript and protein levels [3–5], while there is lack of experimental data on the effect of length of storage in liquid nitrogen of sperm. To improve the knowledge on this issue, we performed a molecular profiling of thawed sperm after 1 week and at least 7 years of storage in liquid nitrogen. For the first time we demonstrated that the length of storage does not dramatically induce changes in the abundance of the human sperm transcriptome. Only five transcripts were more abundant in sperm after a long-time storage in liquid nitrogen, without any effect on the pathways directly involved in the sperm physiology and fertility. This finding is consistent with clinical studies according to long-time storage of sperm does not affect post-thaw clinical and obstetric outcomes [9].

Our discovery of non-harmful effects of storage time on the sperm transcriptome profile suggests that the developmental potential of frozen/thawed spermatozoa is independent of the duration of their conservation in liquid nitrogen. The wider implication of our novel and reassuring finding is noteworthy for the safety of long-term sperm banking, in particular in oncofertility.

Although the statistical analysis was robust, the result of five more abundant transcripts after long-term storage is biologically interpretable considering that they could be expressed at a higher level before freezing. Of course, the important message of this study, namely that the duration of cryopreservation does not adversely affect the semen, does not change, but it should be considered that men who have stored their sperm for a long time had cryopreserved for health problems. Specifically, 73% (8/11) of these men had a malignant disease. Some studies have reported poor sperm quality in cancer patients, with a cause not well understood [20]. It is noteworthy that functionally the most abundant transcripts after long-term storage are involved in methylation, degradation of mRNA and proteins, and spermatogenesis, all processes that could be involved in the dysregulation of sperm function associated with pathological conditions. In particular, the protein encoded by *DCP1A* is the catalytic component of the decapping complex that removes the 7-methyl guanine cap structure from mRNA molecules, yielding a 5’-phosphorylated mRNA fragment and a 7-methylguanosine diphosphate. This is an early crucial step in the process of mRNA degradation in both normal mRNA turnover and in nonsense-mediated mRNA decay since after the 5’ cap is removed the mRNA is vulnerable to attack by exonucleases. *MSI2* encodes an RNA binding protein that regulates mRNA translation and stability. It is as a crucial factor in the regulation of gene expression during spermatogenesis contributing to the proper self-renewal and differentiation of spermatogonial stem cells [21]. Two transgenic mouse models with germ cell-specific overexpression of *MSI1* or *MSI2* transcripts showed a significant decrease in the ability of sperm to bind effectively to the zona pellucida of a control mouse oocyte, with *MSI2* overexpression resulting in male infertility [21].

Regarding *HUWE1* transcript, it encodes an E3 ubiquitin ligase which mediates ubiquitination and subsequent proteasomal degradation of target proteins in various processes including spermatogenesis [22].

FAM217A is highly expressed in testis and interacts with sperm associated antigen 6 (SPAG6). Studies in mice suggest that this protein complex is involved in sperm flagellar motility and maintenance of the structural integrity of mature sperm [23]. Dysregulation of this complex may have a role in blocking fertilization.

The protein encoded by *PCSK1N* gene functions as an inhibitor of prohormone convertase 1, which regulates the proteolytic cleavage of neuroendocrine peptide precursors. To the best of our knowledge there are no data that can suggest thoughts and considerations on its involvement in sperm function.

Based on our findings, we argue that storage in liquid nitrogen does not damage sperm function or worsen it. At the same time, we have to consider the open question of possible epigenetic damage transmissible to embryos obtained from frozen sperm and the long-term offspring phenotype. It is known that reactive oxygen species production during the freezing–thawing process is associated with elevated DNA damage and epigenetic changes [16]. The most investigated epigenetic process is DNA methylation that consists in the addition of methyl groups to cytosine or adenine bases of DNA in CG dinucleotide context (CpG sites). These epigenetic modifications are maintained throughout cell divisions by DNA methyltransferases. DNA methylation is correlated with gene silencing and other regulatory mechanisms such as imprinting or X-chromosome inactivation and silencing of centromeric sequences. Additional epigenetic regulations comprise post-transcriptional histone modifications, including acetylation, methylation, phosphorylation, and glycosylation ubiquitination. Several studies have investigated the impact of human sperm cryopreservation on epigenetic markers. Although a number of studies reported that cryopreservation did not affect the level of global DNA methylation of human sperm [24], contradictory results have reported the effect of sperm cryopreservation on epigenetic changes [25]. The discrepancy between findings from these studies may be due to the small sample size, various semen parameters, different methods used for the evaluation of sperm DNA damage, different protocols for sperm freezing–thawing, and cryoprotectant agents Oxidative stress in sperm can influence epigenetic reprogramming during early embryonic development [26], although in a study by Chao and colleagues the epigenetic reprogramming of mouse embryos derived from cryopreserved spermatozoa was similar to that of embryos derived from fresh spermatozoa [27]. In human, despite concern about damage to sperm nuclear DNA during cryopreservation, there is little information about the effects of cryopreservation on the health of children born with frozen spermatozoa and no confirmed increase in genetic or phenotypic anomalies in offspring has been recognized. Therefore, multicenter studies with extended follow up of offspring under the same conditions of cryopreservation and DNA methylation analysis are needed to make any definitive conclusion about the effect of the cryopreservation process on sperm epigenetic damages.

We are aware that this study has some limitations due mainly to the lack of data validation in a wider sample cohort and by quantitative PCR and/or at protein level, although we have applied strict criteria for gene selection (FDR = 0). In a future study it would also be interesting to integrate the transcriptome data with the examination of sperm characteristics of warmed cryopreserved aliquots. Further research should be undertaken to include in analyses “older” frozen sperm, cryopreserved semen with substandard quality (i.e., oligozoospermic, asthenozoospermic or teratozoospermic samples), as well as to test the effects of various sperm cryopreservation protocols.

## Conclusions

This paper for the first time showed that the length of storage of human sperm in liquid nitrogen does not alter critical pathways involved in sperm physiology and fertility. It follows that the potential damage produced to frozen/thawed spermatozoa is only due to the cryopreservation procedure, rather than the storage time per se. These findings are noteworthy for the issue of long-term sperm banking safety, i.e. fertility preservation, gamete donation.

## Supplementary Information

Supplementary Material 1.

## Data Availability

No datasets were generated or analysed during the current study.

## Abbreviations

ART: Assisted Reproduction Technology
BP: Biological Process
CC: Cellular Components
DAVID: Database for Annotation, Visualization, and Integrated Discovery
FDR: False Discovery Rate
FE: Feature Extraction
GEO: Gene Expression Omnibus
GO: Gene Ontology
ICSI: Intracytoplasmic Sperm Injection
IUI: Intrauterine Insemination
KEGG: Kyoto Encyclopedia of Genes and Genomes
MF: Molecular Function
ROS: Reactive Oxygen Species
SAM: Significance Analysis of Microarrays
