# Supplementary material for: The storage time of cryopreserved human spermatozoa does not affect pathways involved in fertility
Source: Basic Clin Androl. 2024 Sep 17;34:15. doi: 10.1186/s12610-024-00231-4 (PMC11406918; doi:10.1186/s12610-024-00231-4)
Supplement: Supplementary file 1 — Supplementary Material 1. [file 12610_2024_231_MOESM1_ESM.docx]

**Supplementary Table 1.** Semen parameters and clinical data of donors enrolled in the study.

| **ID** ^a^ | **Age** ^b^ **(years)** | **Semen parameters** | | | | **Length of cryoconservation** | **History of men** | |
| --- | --- | --- | --- | --- | --- | --- | --- | --- |
|  |  | **Volume (ml)** | **Total sperm number (mil)** | **Total motility (%)** | **Progressive motility (%)** |  | **Reason for cryopreservation** | **Fertility status** ^c^ |
| 1S | 44 | 4.5 | 324 | 70 | 60 | 1 week | free choice to participate in the study | normozoospermic |
| 2S | 34 | 4.0 | 276 | 55 | 60 | 1 week | free choice to participate in the study | normozoospermic |
| 4S | 29 | 2.5 | 290 | 75 | 70 | 1 week | free choice to participate in the study | children |
| 9S | 33 | 3.4 | 75 | 40 | 35 | 1 week | free choice to participate in the study | children |
| 10S | 46 | 1.5 | 41 | 60 | 40 | 1 week | free choice to participate in the study | children |
| 11S | 35 | 5.0 | 630 | 65 | 55 | 1 week | free choice to participate in the study | children |
| 12S | 42 | 3.5 | 479 | 70 | 60 | 1 week | free choice to participate in the study | normozoospermic, partner of an infertile woman |
| 13S | 39 | 2.0 | 166 | 60 | 50 | 1 week | free choice to participate in the study | children |
| 16S | 33 | 3.0 | 12 | 60 | 60 | 1 week | free choice to participate in the study | children |
| 17S | 45 | 4.0 | 284 | 55 | 55 | 1 week | free choice to participate in the study | children |
| 18S | 37 | 4.5 | 283 | 72 | 57 | 1 week | free choice to participate in the study | children |
| 19S | 34 | 1.5 | 35 | 55 | 55 | 1 week | free choice to participate in the study | children |
| 20S | 34 | 3.0 | 57 | 65 | 55 | 1 week | free choice to participate in the study | normozoospermic |
| 366L | 22 | 3.7 | 96 | 70 | 56 | 10 years | Hodgkin's lymphoma | normozoospermic |
| 451L | 26 | 3.2 | 122 | 47 | 39 | 9 years | gastric lymphoma | normozoospermic |
| 466L | 31 | 9.0 | 108 | 85 | 77 | 9 years | testicular cancer | children |
| 507L | 46 | 5.5 | 214 | 70 | 55 | 9 years | induratio penis plastic | normozoospermic |
| 511L | 35 | 2.2 | 90 | 43 | 39 | 9 years | brain tumor | normozoospermic |
| 513L | 24 | 3.4 | 306 | 65 | 45 | 9 years | gender dysphoria | normozoospermic |
| 515L | 45 | 3.0 | 116 | 85 | 78 | 9 years | myelofibrosis | normozoospermic |
| 517L | 26 | 3.2 | 61 | 58 | 42 | 8 years | testicular cancer | normozoospermic |
| 575L | 27 | 5.5 | 643 | 45 | 30 | 8 years | Hodgkin's lymphoma | children |
| 600L | 35 | 4.5 | 184 | 75 | 65 | 7 years | Hodgkin's lymphoma | normozoospermic |
| 658L | 39 | 3.0 | 102 | 48 | 45 | 7 years | testicular cancer | children |

**Legend.** The Table shows semen parameters at the time of freezing and clinical data of donors enrolled in the study. ^a^: identification code of each sample; ^b^: age of donors at the time of semen freezing; ^c^: fertility status of donors, considering their potential (normozoospermia) or proved fertility (if men had children at the time of semen freezing).
